# Impact of a ‘Catheter Bundle’ on Infection Rates and Economic Costs in the Intensive Care Unit: A Retrospective Cohort Study

**DOI:** 10.3390/nursrep14030145

**Published:** 2024-08-09

**Authors:** Alberto Lucchini, Marco Giani, Emanuele Rezoagli, Giulia Favata, Annagiulia Andreani, Marta Spada, Luigi Cannizzo, Nicola Barreca, Matteo Cesana, Stefano Citterio, Stefano Elli

**Affiliations:** 1 General Adult and Paediatric Intensive Care Unit, Fondazione IRCCS San Gerardo dei Tintori, Via Pergolesi 33, 20900 Monza, Italy; marco.giani@unimib.it (M.G.); emanuele.rezoagli@unimib.it (E.R.); luigi.cannizzo@irccs-sangerardo.it (L.C.); nicola.barreca@irccs-sangerardo.it (N.B.); 2 Medicine and Surgery Department, University of Milano-Bicocca, 20126 Milan, Italy; 3 Direction of Health and Social Professions, Fondazione IRCCS San Gerardo dei Tintori, 20900 Monza, Italy; matteo.cesana@irccs-sangerardo.it (M.C.); stefano.citterio@irccs-sangerardo.it (S.C.); stefano.elli@unimib.it (S.E.); 4 Critical Care Nursing, University of Milano-Bicocca, 20126 Milan, Italy; giuli.favata@gmail.com (G.F.); annagiulia.a@hotmail.it (A.A.); marta.spada95@libero.it (M.S.)

**Keywords:** CRBSI, infection prevention, bundle, port protector, needle-free, CLABSI

## Abstract

Introduction: Catheter-related infections (CBRSIs) are a widespread problem that increase morbidity and mortality in intensive care unit (ICU) patients and management costs. Objective: The main aim of this study was to assess the prevalence of CBRSIs in an intensive care unit following international literature guidelines for managing vascular lines in critically ill patients. These guidelines include changing vascular lines every 7 days, using needle-free devices and port protectors, standardising closed infusion lines, employing chlorhexidine-impregnated dressings, and utilising sutureless devices for catheter securement. Materials and Methods: This single-centre retrospective observational study was conducted in a general Italian ICU. This study included all eligible patients aged > 1 year who were admitted between January 2018 and December 2022. Results: During the study period, 1240 patients were enrolled, of whom 9 were diagnosed with a CRBSI. The infection rate per 1000 catheters/day was as follows: femorally inserted central catheter, 1.04; centrally inserted central catheter, 0.77; pulmonary arterial catheter 0.71, arterial catheter, 0.1; and peripherally inserted central catheter and continuous veno-venous haemodialysis dialysis catheters equal to 0. No difference in CRBSI was observed between the years included in the study (*p* = 0.874). The multivariate analysis showed an association between the diagnosis of CBRSI and Nursing Activities Score (per single point increase β = 0.04–95%CI: −0.01–0.09, *p* = 0.048), reason for ICU admission—trauma (β = 0.77–95%CI: −0.03–1.49, *p* = 0.039), and use of therapeutic hypothermia (β = 2.06, 95%CI: 0.51–3.20, *p* < 0.001). Implementing the study protocol revealed a cost of EUR 130.00/patient, equivalent to a daily cost of EUR 15.20 per patient. Conclusions: This study highlights the importance of implementing a catheter care bundle to minimise the risk of CRBSI and the associated costs in the ICU setting. A policy change for infusion set replacement every 7 days has helped to maintain the CRBSI rate below the recommended rate, resulting in significant cost reduction and reduced production of ICU waste

## 1. Introduction

Catheter-related bloodstream infections (CRBSIs) represent a significant and persistent challenge in intensive care units (ICUs) worldwide [1,2,3]. These infections are the leading cause of illness and fatality among critically ill patients, often resulting in extended hospital stays, increased healthcare expenses, and considerable patient suffering [1]. The occurrence of CRBSIs involves colonisation of both the internal and external surfaces of catheters by microorganisms, which results in the spread of these pathogens throughout the bloodstream [2,4]. Despite advancements in infection control practices and catheter technology, CRBSIs continue to pose a significant threat to the ICU environment, necessitating constant vigilance and innovative preventive measures [1].

The use of intravascular catheters (arterial and/or venous) is ubiquitous in ICUs, where patients frequently require intensive monitoring and support, including the administration of vasoactive drugs, medications, fluids, blood products, and parenteral nutrition. Centrally inserted central catheters (CICCs), peripherally inserted central catheters (PICCs), pulmonary artery catheters (PACs), continuous veno-venous haemofiltration (CVVH) catheters, and radial or femoral artery catheters are important resources for treating critically ill patients [5,6,7]. These vascular catheters provide consistent and reliable monitoring and access for delivery of life-saving therapies. The continued use of these devices undermines the integrity of the skin, which serves as the body’s main defence against infection, thereby providing a gateway for pathogens to enter. Pathogens primarily linked to CRBSIs comprise mainly Gram-positive bacteria, including coagulase-negative staphylococci and Staphylococcus aureus, as well as Gram-negative bacteria and fungi, particularly Candida species [3,8,9]. The origins of these pathogens are diverse and can stem from the patient’s skin bacteria, catheter hub contamination, and the hands of healthcare workers. The chance of acquiring CRBSIs depends on several factors including the type of catheter used, duration for which the catheter is left in place, location of the exit site, and the patient’s general health condition [1,3,9]. The prevention of CRBSIs is a multifaceted challenge that involves stringent adherence to aseptic techniques during catheter insertion and maintenance, use of antimicrobial-impregnated catheters, and implementation of evidence-based care bundles [1,9]. Bundles of care, which comprise evidence-based practices that have been proven to enhance patient outcomes when used together, are essential for reducing the prevalence of CRBSIs [10,11]. These bundles are generally divided into two areas: bundles for device placement (e.g., maximal sterile barrier precautions during central venous catheter insertion, use of a 2% chlorhexidine (CHG) preparation for skin antisepsis, use of sutureless devices for catheter securement, use of polyurethane transparent dressing for exit site protection, selection of the optimal emergency site, tunnelling of the catheter when necessary [12,13,14,15], no routine replacement of central venous catheters for prevention of infection, use of a 2% chlorhexidine wash for daily skin cleansing), and bundles for daily management of infusion and monitoring systems (e.g., dressing change and line replacement every 7 days, application of chlorhexidine-impregnated dressing, employment of needle-free connectors, utilisation of port protectors, implementation of standardised, non-modifiable hub) [1,9]. Despite the implementation of established protocols to prevent CRBSIs, the infection rates continue to vary among ICUs. When the risk was expressed as CRBSIs per 1000 intravascular devices rates, the highest rates in ICU patients occurred with CVVH catheters (4.8), PACs (3.7), non-medicated CICCs (2.7), arterial catheters for haemodynamic monitoring (1.7), medicated-chlorhexidine-silver-sulfadiazine CICCs (1.6), and PICCs (1.1) [2]. Recently, several studies have revealed a significant increase in the occurrence of CRBSIs during the COVID-19 pandemic [6,7,16]. The frequency of these infections tripled during the study period. A variety of factors have contributed to this increase, including the increased workload for nurses and patients receiving large doses of steroids, the increased use of non-tunnelled femoral catheters for central line availability, or CVVH procedures [17,18,19], most of whom were positioned in a prone position. The use of this position poses challenges for both the insertion and care of endovascular catheters [20]. CRBSIs not only have a profound impact on health, but also on the economy. The expenses incurred are not limited to the extended duration of ICU stay and additional treatments are needed to manage infections [1,3]. They also encompass broader repercussions including increased sickness and fatality rates.

The aim of this study was to investigate the prevalence of CRBSIs in a cohort of critically ill patients admitted to a general intensive care unit in Italy over a 5-year period. The secondary aim was to determine the cost per day of implementing a specialised care bundle for daily management of vascular lines.

## 2. Materials and Methods

### 2.1. Study Design and Setting

A retrospective analysis was conducted at an Italian Extracorporeal Membrane Oxygenation centre to determine the occurrence and features of CRBSIs in patients admitted between 1 January 2018 and 31 December 2022. In the preceding period, from 2014 to 2017, the incidence of CRBSI in the ICU for all types of vascular catheters (CICCs, FICCs, PACs, CVVHs, and arterial catheters) was 0.53 per 1000 catheter days. A total of 16 cases of CRBSI were reported out of 29,980 catheter days. Before the COVID-19 pandemic, the intensive care unit (ICU) had a capacity of ten beds. During the initial COVID-19 wave, from February to May 2020, the number of ICU beds increased to 21, and during the second wave, from 20 October to 21 January the number of beds increased to 19. During the study period, the nurse-to-patient ratio was in the following range: 0.5 (COVID-19 pandemic waves) to 0.6 (before and after pandemic). The hospital has a structured surveillance program for the correct hand hygiene application, managed by the infection prevention office, with monthly assessments. During the study period, the compliance of ICU operators was found to be consistently above the minimum recommended level by the Word Health Organization (>75%). During the COVID period (February 2020–December 2021), assessments were conducted on a quarterly basis. This study was conducted in accordance with the Strengthening the Reporting of Observational Studies in Epidemiology (STROBE) guidelines.

### 2.2. Inclusion Criteria

All eligible patients, aged > 1 year, with complete electronic medical records containing information about one of the following devices during the study period were included in the analysis—CICC, PICC, PAC, CVVH catheter, femoral inserted central catheter (FICC), and arterial catheters for haemodynamic monitoring. Patients with missing or incomplete data on CRBSIs and vascular access management were excluded from this study. Patients who were admitted to the intensive care unit for monitoring after elective surgery and managed in a dedicated room with an ICU stay of less than 24 h were excluded from the study.

### 2.3. Protocol

The CICC and FICC implanted during the study period were coated with chlorhexidine silver-sulfadiazine [1]. The following recommendations were adopted for all enrolled patients during the study period.

Before catheter insertion: daily bathing in patients aged > 6 months with chlorhexidine 4% preparation [1,3,9].

During catheter insertion, hand hygiene prior to catheter insertion or manipulation, an all-inclusive catheter kit, ultrasound guidance for catheter insertion, maximum sterile barrier precautions during catheter insertion, and 70% isopropyl alcohol chlorhexidine (2%) were used for skin preparation [1,3].

After catheter insertion, chlorhexidine-containing dressings for all managed catheters in patients over 6 months of age, dressing replacement, and site care with a chlorhexidine-based antiseptic at least every 7 days, or immediately if the dressing is soiled, loose, or damp, use of closed infusion line with neutral needle-free connectors (pre-assembled standardised hubs), use of port protectors (disposable passive disinfection device containing 70% isopropyl alcohol), routine replacement of administration sets not used for blood, blood products, or lipid formulations every 7 days; double securement of all vascular catheters with two sutureless devices; linear 250 cm syringe pump extension with needle-free connector on the syringe side [1,4,21,22,23,24,25].

Daily compilation of a specific form in the clinical computer system with reminders for the points specified in the bundle. Monthly verification of device usage through the hospital consumption monitoring system.

**Figure 1:**
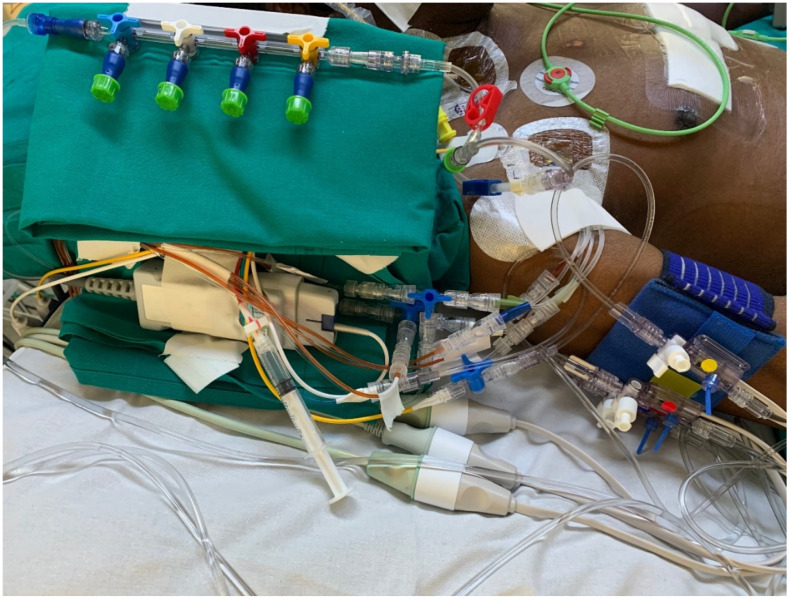
Infusion and monitoring lines during the study period.

**Figure 2:**
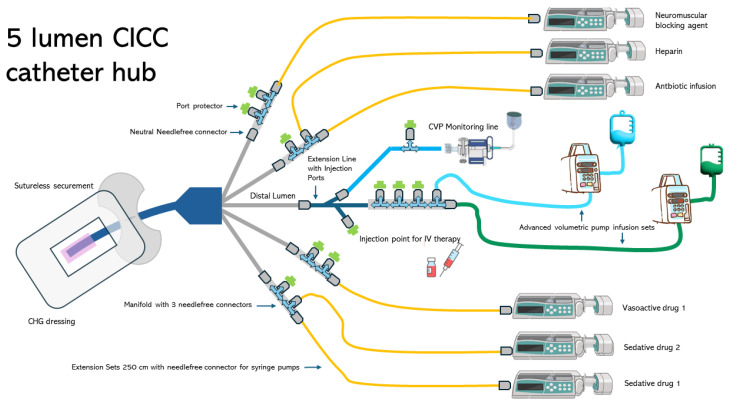
Design of the infusion line used during the study.

Figure 1 and Figure 2 illustrate examples of the strategies applied in the management of the infusion line during the study period.

### 2.4. Data Collection

Electronic medical records were retrospectively examined to collect the following data: CRBSI diagnosis, catheter characteristics, daily management of the vascular line, and demographic information. All parameters were recorded using Drager Medical Innovian Suite patient management system software (Innovian Medical Suite© Drager Medical—version VF 7.0.1, Lubeck, Germany). In accordance with current guidelines, CRBSIs have been assessed based on paired blood cultures considering the differential time to positivity > 2 h or, in cases of persistent withdrawal occlusion, based on the positive culture of the catheter tip after removal [23,26]. Diagnostic procedures for identifying CRBSI are initiated when clinical signs of infection are present or suspected by clinicians. Two sets of blood cultures, each containing at least 20 mL of blood, were collected—one from a peripheral vein and one from the catheter after the removal of the needle-free connector placed on the catheter lumen. If there were local signs of infection, the catheter was immediately removed and replaced at another site if the patient’s clinical needs required it. If there were no local signs of infection, no haemodynamic instability (drop in systolic blood pressure and/or need for catecholamines), and no signs of acute sepsis with a significant rise in temperature, the catheter was left in place, and antibiotic therapy was evaluated. If the preliminary report (within 24–48 h) of the blood culture was positive, the catheter was removed and replaced at a different site. If the preliminary report was negative, the final result was awaited. If the final result was positive, the catheter was removed and replaced at a different site.

### 2.5. Data Analysis 

Shapiro–Wilk’s test and visual inspection were used to test for normality of distribution. Categorical variables are presented as counts (proportions), whereas continuous variables are expressed as means (standard deviations). CRBSI rates were expressed as the number of infections per 1000 catheter days, according to the recommendations of the Centers for Disease Control and Prevention. Categorical variables were compared between the two groups (patients with and without CRBSIs) using either Pearson’s chi-squared test or Fisher’s exact test, as appropriate [3]. Multivariate analysis was conducted to determine the independent association between CRBSI diagnosis and investigated factors. The results are reported as estimates with 95% confidence intervals (CI) and corresponding *p*-values. Statistical significance was set at *p* < 0.05. Statistical analysis was performed using the JMP 15.2 software (SAS Institute, Cary, NC, USA).

### 2.6. Ethics

Data were collected in a retrospective study (OVASC 2—ID 4024\_S\_M) approved by the local ethics committee (Comitato Etico Territoriale Lombardia 3) on 13 December 2023.

## 3. Results

During the study period, 1631 patients were admitted to the ICU. A total of 384 elective postoperative patients were excluded from this study. Data were not available for seven patients. A total of 1240 patients met all the inclusion criteria and were included in the study. The mean age was 57.42 years (±19.80), 448 (36%) were female and the mean ICU length of stay was 9.01 days (±12.35). In total, 1092 patients (88%) survived and were discharged from the ICU. CRBSI was diagnosed in nine patients using 10 different vascular catheters. The demographic and clinical characteristics of the enrolled patients are presented in Table 1. 

The logistic model (Degree of Freedom: 5, chi-square: 17.560—*p* = 0.004) showed an association between the diagnosis of CBRSI and Nursing Activities Score (per single point increase β = 0.04, 95%CI: −0.01–0.09, *p* = 0.048), reason for ICU admission—trauma (β = 0.77, 95%CI: −0.03–1.49, *p* = 0.039), and use of therapeutic hypothermia (β = 2.06, 95%CI: 0.51–3.20, *p* < 0.001). There was no association between the diagnosis of CRBSI and other variables such as the use of mechanical ventilation (*p* = 0.997) and length of ICU stay (*p* = 0.074).

Table 2 presents the pathogens detected in the nine patients with confirmed CRBSI, along with the affected catheters. 

**Table 2:** Pathogens responsible for CRBSI in the 5 years covered by the study. Legend: CICC: centrally inserted central catheters; FICC: femorally inserted central catheters; PAC: pulmonary artery catheters.

Patient	Year	Alive	ECMO	COVID-19	Catheter Type—1	Pathogen	Catheter Type—2	Pathogen	Antibiotic Therapy (all ICU Stay)	Time in ICU before CRBSI	Time in ICU after CRBSI
1	2018	Yes	Yes	No	CICC	Escherichia coli Extended Spectrum Beta-Lactamase (ESBL)	PAC	Staphylococcus hominis	Linezolid Meropenem Amikacin Levofloxacin Ciprofloxacin Tazobactam/piperacillin	19	7
2	2018	No	No	No	CICC	Enterococcus faecium			Linezolid Amphotericin B Meropenem Acyclovir	12	5
3	2020	Yes	No	No	FICC	Bacillus cereus			Tazobactam/piperacillin Metronidazole Daptomicina		9
4	2020	Yes	No	Yes	CICC	Enterococcus faecalis			Anidulafungin Micafungin Meropenem Linezolid Tazobactam/piperacillin	5	16
5	2021	Yes	No	Yes	CICC	Burulderia			Meropenem Linezolid Tazobactam/piperacillin Levofloxacin	15	20
6	2021	Yes	No	No	CICC	Stenotrophomonas maltophilia			Tazobactam/piperacillin Cefazolin Gentamicin	4	21
7	2021	Yes	No	Yes	CICC	Candida albicans			Levofloxacin Tazobactam/piperacillin Metronidazole	7	11
8	2022	Yes	No	Yes	Arterial catheter	Morganella morganii			Ampicillin Daptomycin Tigecycline	11	8
9	2022	Yes	No	No	CICC	Klebsiella oxytoca			Tazobactam/piperacillin Amoxicillin/clavulanic acid	7	6

No statistical difference in the incidence of CRBSI was observed among the years included in the study (*p* = 0.874). During the study period, 3128 catheters were implanted, resulting in a total catheter exposure of 24.776 days. The overall incidence of CRBSI in the enrolled population was 0.4 per 1000 catheter days. As reported in Table 3, the incidence of CRBSI per 1000 catheter days was equal to zero for PICC and CVVH catheters, 0.1 for arterial catheters, 0.71 PAC, 0.77 for CICC, and 1.04 FICC. Regarding CICC and FICC, it was found that out of the 1229 catheters used in the study period, 467 (38%) were five-lumen catheters, 246 (20%) four-lumen catheters, 246 (20%) three-lumen catheters, 111 (9%) two-lumen catheters, 80 (6%) seven-lumen catheters, and 80 (6%) were a single-lumen catheter.

**Table 3:** CRBSI infection rate per all catheters used in the study period. Legend: femorally inserted central catheter (FICC); centrally inserted central catheter (CICC); pulmonary artery catheter (PAC); peripherally inserted central catheter (PICC); continuous veno-venous haemofiltration (CVVH).

	n (%)	Mean (SD) Catheter Days Placement	Cumlative Catheter Days	Confirmed CRBSI	Incidence CRBSI/1000 Cath. Days
FICC	261 (21%)	4 (±4)	965	1	1.04
CICC	968 (78%)	9 (±11)	9053	7	0.77
PAC	145 (12%)	10 (±8)	1417	1	0.71
Arterial catheter	1204 (97%)	8 (±10)	9747	1	0.10
PICC	362 (29%)	5 (±7)	1920	0	0
CVVH catheter	188 (15%)	9 (±11)	1674	0	0

The total cost of all devices (excluding catheter cost) used for managing vascular lines following catheter implantation was EUR 279,104.00, resulting in a cost of EUR 130.00, equivalent to a daily cost of EUR 15.20 per patient. A summary of the devices used in this study is provided in Table 4.

## 4. Discussion

The primary objective of this study was to examine the incidence and risk factors of CRBSI in patients admitted to an Italian general ICU over a 5-year period. The incidence of CRBSI was found to be 0.4 per 1000 catheter days across all devices, which is lower than the rate reported in previous studies [2,3] and the rate reported in non-ICU patients for CICCs and PICCs [27]. The rate of CRBSI was found to be lower than the previous recorded data in the ICU over the 4 years prior to the study (0.53/1000 catheter days). Furthermore, despite literature data showing an increase in the rate of CRBSIs during the pandemic, no statistically significant differences were observed during the study years (*p* = 0.874) [6,16]. Within this sample, the highest rate of CLABSI was observed for FICC catheters (1.04), followed by CICC catheters (0.77). CRBSI was diagnosed in nine patients and involved ten different catheters (eight CICC, one FICC, one PAC, and one arterial catheter). 

The femoral vein is more likely to develop CRBSI than the internal jugular or subclavian veins [3]. Templeton et al. identified the number of lumens as an independent risk factor for CRBSI [28]. Patients using multiple-lumen catheters have a 3.41-fold increased risk of developing CRBSIs compared with those using single-lumen catheters [29]. A single-lumen catheter may be the best option for intravenous infusion, and reducing the number of lumens would be beneficial. However, clinical need requires the ability to manage multiple infusions in patients admitted to the ICU. The issue of an adequate number of venous lumens concerns the compatibility of drugs that need to be infused via the same route, differentiation of flow problems when two drugs are infused through the same lumen, and haemodynamic impact related to the management of vasoactive drugs (norepinephrine, adrenaline, dobutamine, dopamine, etc.) [30,31,32]. In this study, 64% of patients with CICC/FICC had at least four lumens. Despite the use of many lumens, the CRBSI rate was below the recommended limits specified in the literature. Therefore, the catheter management protocol described in this study may play a crucial role in achieving this objective. Standardising venous lines (Figure 1 and Figure 2) using predefined hubs (manifold needle-free connectors, three-way stopcocks with needle-free connectors, and dedicated extension lines for administering therapy at specific times) aimed to reduce the number of overall manipulations by nurses and physicians. Three-way stopcocks and connectors are generally the most commonly colonised components of infusion sets [1,3,29]. The use of predefined hubs without the need to add or remove three-way stopcocks when continuous infusions are suspended and/or resumed is one of the recommendations suggested in the literature to reduce the risk of CRBSI development [1,3]. In addition, the extensive use of needle-free connectors has been associated with the use of port protector systems [4,22,33]. Pathogens may gain access to the bloodstream through either an extraluminal or intraluminal pathway and generate CRBSI [29]. The intraluminal pathway is the most common cause of infection and is often caused by improper antiseptic catheter-handling procedures. This pathway begins at the catheter hub and needleless connectors [22,33]. To prevent intraluminal contamination, it is crucial, if a closed line is implemented, that needleless connectors are scrubbed with a disinfectant wipe and then dried before using the catheter [1,3,9]. However, inadequate disinfection increases the risk of CLABSIs [34]. Port protectors have been developed to decrease CLABSIs by reducing the effects of variations in scrubbing duration and techniques [4,22]. Port protectors typically contain a disinfectant, usually 70% isopropyl alcohol or chlorhexidine gluconate, which continuously bathes the access point in an antimicrobial agent when the cap is screwed directly onto a needleless connector. Port protectors can be left in place between infusions, providing improved disinfection and protection against touch or airborne pathogens invading the hub [33]. As long as the port protector remains in place, the needleless connector remains inaccessible, disinfected, and protected for up to 7 days. The latest clinical research endorses the utility of port protectors for the passive and continuous disinfection of needleless connectors without the need for more time-consuming manual cleansing of the hub [4,22,33]. This topic is of great significance because, as the nursing workload increases or the nurse-to-patient ratio declines, the likelihood of acquiring CRBSI increases [1,3,19]. Even in the population enrolled in this study, there was an association between the diagnosis of CBRSI and nursing workload expressed by the nursing activities score (per single point increase β = 0.04–95%CI: −0.01–0.09, *p* = 0.048). During the study period, which encompassed the first and second waves of COVID-19 in Italy, an increase in the rate of CRBSIs was observed both within and outside the country [6,16]. Additionally, numerous reports have highlighted the increased workload of nurses during pandemic [17,18]. Despite these two risk factors, no statistically significant differences were found in the incidence of CRBSI, divided by year, in the sample analysed in this study from 2018 to 2022 (*p* = 0.874).

During the study period, the implemented protocol adhered to two other recommendations: employing chlorhexidine-containing dressings and regularly replacing administration sets every 7 days [1,21,25]. Since 2017, the United States Centers for Disease Control and Prevention (CDC) has recommended the use of CHG-impregnated dressings in routine applications [3]. A recent systematic review and meta-analysis confirmed that CHG-impregnated dressings are effective in preventing intravascular catheter infections [25]. A CHG gel dressing was used in this study. Compared with CHG-impregnated sponges, gel-dressing technology allows continuous monitoring, which may aid clinicians in detecting early signs of inflammation at the insertion site. In addition, CHG dressing was coupled with a suture-free system to secure all catheters used in this study. Sutures may serve as a nidus for bacterial colonisation. Dressing and sutureless devices were changed every 7 days or immediately if the dressing was soiled, loose, or damp. Routine replacement of invasive pressure monitoring lines and administration sets not used for blood, blood products, or lipid formulations was performed at intervals of 7 days, as suggested in recent studies [35,36]. Reducing the frequency to once a week reduces the number of manipulations, nursing workload, and necessary costs. CRBSI costs EUR 42,294.57 (USD 45,814) per case [29]. 

Starting with the observation of the cost of a single CRBSI (approximately EUR 40,000), it is possible to compare the effect of some of the items in the bundle used in the ICU. For example, the cost of implementing port protectors has generated an annual expense during the study period of around EUR 6000. The reduction of one case of CRBSI per year generates economic savings that can support the implementation of both port protectors and a completely closed infusion line (needle-free connectors, preassembled ramps, etc.). At the time of economic resource reduction in healthcare in Europe, the possibility of verifying the impact on cost reduction, determined by the reduction in the incidence of CRBSI, should always be considered. Of course, the introduction of bundles with the introduction of new devices necessarily involves an increase in the costs to be borne during the programme’s startup period. However, as analysed in this study over a 5-year period, it is possible to see how the expenses incurred amortise within 60 months.

In a sample of 1240 patients, the total cost of all devices used for managing vascular lines during indwelling time was EUR 270,104, resulting in a cost of EUR 130, equivalent to a daily cost of EUR 15.20 per patient. However, limited data were available to compare the costs recorded in this study. Daily costs do not include delivered drugs. Rickard et al. in 2021, estimated that 7-day rather than 4-day replacement of infusion sets will reduce EUR 313 per central venous access device [36]. These costs include staff time and consumables from infusion set replacement procedures; however, it is unclear whether drug costs were included in their calculations. Van de Pol and colleagues recently reported in a before-after study in 1409 patients, that 7-day versus 4-day replacement, saved 260 nursing hours were and estimate of EUR 17,250 saved, based on a cost price of EUR 50 per replacement set [37]. 

It is crucial to emphasise that ICUs generate significant amounts of waste and that each day, ICU care produces seven bags of waste per patient. According to the European Federation of Critical Care Nursing associations’ position statement, “Towards sustainable intensive care”, waste reduction can be achieved by adjusting the replacement frequencies of materials [38]. Implementing a 7-day strategy to replace vascular device lines does not increase the incidence of CRBSI, thus decreasing the use of disposable materials and directly affecting plastic consumption and pollution [36,37,39].

Finally, the significance of implementing a “catheter bundle” remains a subject of debate. Nevertheless, the findings of this study indicate that, during a 5-year period that included a pandemic that severely tested healthcare systems, applying all interventions supported by available literature can help to keep the rate of CRBSI at the recommended low level. 

The Italian Group of Venous Access Devices (GAVeCeLT) has recently made the decision to provide an updated compendium that summarizes the primary strategies, both conventional and innovative, which are useful for reducing catheter-related complications in the critically ill adult patient [40]. The final document presents statements that answer to four major sets of questions regarding central venous access in the critically ill: 1—before insertion, 2—during insertion, 3—after insertion, and 4—at removal. All the recommendations related to daily management of vascular access are present in the investigated bundle presented in this study. Adopting a specific post-insertion bundle that includes chlorhexidine-containing dressings, dressing replacement and site care every 7 days, closed infusion line with neutral needle-free connectors, and using port protectors, as well as routinely replacing administration sets every 7 days, can help maintain lower rates of CRBSIs in patients admitted to general ICUs, as recommended. Additionally, our study showed how a structured bundle can be implemented with a daily cost per patient of EUR 15.20. In a time when we are called to provide the best care for patients, this study demonstrated how we can manage a reduction in CRBSI while achieving two targets: cost-effectiveness and potential waste reduction.

## 5. Limits

This study had certain limitations. First, it was monocentric and retrospective, which led to methodological issues. Second, the study was conducted in an adult and paediatric general ICU. Patients with neurological or cardiovascular issues were not included in the sample (they were managed in other hospital ICUs). Third, we did not differentiate between COVID-19 and non-COVID-19 groups. Instead, we examined only time subdivided by year. Furthermore, the study may be underpowered, primarily because the incidence of CRBSI was very low.

## 6. Conclusions 

Implementing a dedicated “vascular line” bundle in a general ICU setting, based on current literature recommendations, resulted in an incidence of CRBSI below the suggested limits. A modern bundle for managing monitoring and infusion lines should include the use of chlorhexidine-impregnated medications, the implementation of predefined hubs, and the use of needle-free connectors in conjunction with port protectors. The use of multi-lumen central venous catheters, tailored to the clinical needs of patients (ensuring drug compatibility and reducing flow interference in infusions of multiple drugs through a single lumen), did not increase CRBSI rates beyond the thresholds recommended in the literature. Policy change for replacing infusion sets every 7 days helped maintain the CRBSI rate below the recommended limits, resulting in significant cost reduction and decreased ICU waste production.

## Figures and Tables

**Table 1:** Demographic characteristics of enrolled patients. Legend: ICU: intensive care unit; SAPS II: Simplified Acute Physiology Score; SOFA: Sequential Organ Failure Assessment; ECMO: Extracorporeal Membrane Oxygenation.

	All Patients n = 1240	No CRSBI Group n = 1231 (99%)	CRBSI Group n = 9 (1%)	*p* Value
Gender—female	448 (36%)	445 (36%)	3 (33%)	0.861
Age—years	57.42 (±19.8)	57.48 (±19.78)	48.67 (±20.82)	0.193
Weight—Kg	75.43 (±23.83)	75.44 (±23.87)	74.44 (±18.45)	0.909
Body mass index	30.57 (±40.48)	30.61 (±40.63)	26.09 (±5.39)	0.749
Length of ICU stay—days	9.01 (±12.35)	8.92 (±12.33)	21.11 (±8.07)	<0.001
SAPS II Score	31.43 (±15.03)	31.37 (±15.03)	44.33 (±6.43)	0.148
SOFA Score	4.74 (±3.58)	4.73 (±3.58)	7.33 (±1.15)	0.221
COVID-19	324 (26%)	320 (26%)	4 (44%)	0.088
Trauma	146 (12%)	143 (12%)	3 (33%)	0.044
Mechanical ventilation	988 (80%)	966 (78%)	9 (100%)	0.041
Non-invasive ventilation	193 (16%)	192 (16%)	1 (11%)	0.702
Tracheostomy tube	98 (8%)	97 (8%)	1 (11%)	0.782
Prone position	74 (6%)	72 (6%)	2 (22%)	0.118
ECMO	82 (7%)	81 (7%)	1 (11%)	0.593
Vasoactive drug infusion	627 (51%)	621 (50%)	6 (66%)	0.348
Days with vasoactive drug infusion	3.75 (±8.26)	3.71 (±8.26)	8.22 (±7.58)	0.138
Therapeutic hypothermia	6 (0.5%)	5 (0.4%)	1 (11%)	<0.001
Dialisys	66 (5%)	66 (5%)	0 (%)	0.472
Total parenteral nutrition—yes	121 (10%)	120 (10%)	1 (11%)	0.901
ICU survival	1092 (88%)	1084 (88%)	8 (89%)	0.930
Nursing Activities Score (ICU stay)	71.01 (±15.17)	70.91 (±15.16)	83.19 (±11.83)	0.013

Statistically significant differences between patients were found for the following parameters: length of ICU stay (8.92 ± 12.33 in the no CRBSI group vs. 21.11 ± 8.07 in the CRBSI group; *p* < 0.001), admission for trauma (12% vs. 33%, *p* = 0.044), use of mechanical ventilation (78% vs. 100%, *p* = 0.041), use of therapeutic hypothermia (0.004% vs. 11%, *p* < 0.001), and mean Nursing Activities Score (70.91 ± 15.16% vs. 83.19 ± 11.83%, *p* = 0.013).

**Table 4:** Medical devices used during the study.

Disposable Medical Device	Number of Devices Used	Price EUR	Total Cost EUR
Neutral needle-free connector (Microclave^©^ ICU medical)	23,100	0.34	7952.46
Antireflux needle-free connector (Neutron^©^ ICU medical)	830	2.34	1942.20
Extension Line with Injection Ports (ICU Medical)	5350	1.94	10,393.03
Manifold with 3 needle-free connectors (ICU Medical)	4500	5.55	24,971.31
Manifold with 5 needle-free connectors (ICU Medical)	3840	6.75	25,904.26
Stopcock with 2 needle-free connectors (ICU Medical)	11,225	0.20	2300.20
Clear 3 mL Applicator with Sterile Solution (BD ChloraPrep™)	8910	1.03	9202.13
CHG-gel dressings (Tegaderm CHG—3M)	10,020	6.44	64,555.08
Port protector for needle-free connectors (Curos—3M)	125,000	0.21	26,639.34
Port protector for male connectors (Curos—3M)	10,200	0.21	2173.77
Sutureless securement (small)—(Grip-lock Vygon)	2310	0.20	454.43
Sutureless securement (large)—(Grip-lock Vygon)	2980	0.23	683.93
Extension Sets 250 cm with needle-free connector for syringe pumps (ICU-medical)	23,400	1.25	29,154.10
Standard volumetric pump infusion sets (Alaris™—BD)	14,000	2.20	30,800.00
Advanced volumetric pump infusion sets (Alaris™—BD)	1200	2.81	3372.00
Manual cardiac output set for Pulmonary Artery Catheter (CO set© Edwards)	430	7.86	3380.00
Single kit disposable pressure transducers (TruWave©—Edwards)	5700	6.18	35,226.00
		Total cost	279,104.00

## Data Availability

The data presented in this study are available on request from the corresponding author.

## Public Involvement Statement

No public involvement in any aspect of this research.

## Guidelines and Standards Statement

This study was conducted in accordance with Strengthening the Reporting of Observational Studies in Epidemiology (STROBE) guidelines.

## Use of Artificial Intelligence

The authors used Paperpal© to enhance the language of this study, and subsequently reviewed and revised the content as required. They bear responsibility for the content of this publication.
